# Transcriptomic Profiling and Histological Validation of Preputial Fibrosis in Hypospadias

**DOI:** 10.3390/biomedicines13112786

**Published:** 2025-11-14

**Authors:** Yaping Wang, Zhiwei Peng, Yu Ding, Lijun Zhou, Jiacheng Huang, Min Wu, Yiqing Lv, Yichen Huang, Mingming Yu, Fang Chen

**Affiliations:** 1 Department of Urology, Shanghai Children’s Hospital, School of Medicine, Shanghai Jiao Tong University, Shanghai 200062, China; 2 Department of Urology, Shanghai Sixth People’s Hospital, School of Medicine, Shanghai Jiao Tong University, Shanghai 200233, China; 3 Shanghai Eastern Urological Reconstruction and Repair Institute, Shanghai 200233, China

**Keywords:** hypospadias, transcriptomics, prepuce, fibrosis

## Abstract

**Background:** Hypospadias is often associated with abnormal prepuce development. Investigating the differences between the inner prepuce of hypospadias patients and normal controls at the transcriptomic level and histological characteristics helps to reveal the causes of its developmental abnormalities or implement targeted treatments. **Materials and Methods:** Dorsal and ventral inner preputial tissues were collected from 31 hypospadias patients and 21 phimosis children (controls). Differences in gene expression between the two groups were studied via transcriptomic sequencing and enrichment analysis. Corresponding histological features were further validated by histological staining. **Results:** Transcriptomic sequencing results showed that, compared to the control group, the dorsal inner prepuce of the hypospadias group had 97 upregulated and 10 downregulated genes; the ventral prepuce had 140 upregulated and 99 downregulated genes. Among all upregulated genes, 44 were closely related to fibrosis. Other significantly enriched terms included cornified envelope formation, efferocytosis, C-type lectin receptor signaling pathway, and complement and coagulation cascades. Histological validation revealed that the dorsal inner prepuce of hypospadias children contained more collagen fibers, a higher ratio of type I/III collagen, and lower microvessel density, showing some correlation with the severity of hypospadias. **Conclusions:** This study demonstrated a hyper-fibrotic state in the inner prepuce of hypospadias, which may significantly impact post-operative wound healing and complications.

## 1. Introduction

Hypospadias is a common congenital malformation of the male genitourinary system, characterized by abnormal urethral opening, abnormal distribution of the prepuce, and ventral penile curvature. Based on the position of the urethral meatus after straightening, hypospadias can be classified as distal, middle, or proximal. Surgery is the only treatment, but the postoperative complication rate can be over 50% [1]. Complications such as urethrocutaneous fistula, urethral stricture, urethral diverticulum, and recurrent penile curvature are closely related to severity, with the complication rate for proximal hypospadias reaching 57.7% [2]. In contrast, the complication rate for urethral reconstruction surgeries for non-hypospadias reasons (e.g., trauma) is significantly lower, at 5.6–20% [3,4,5]. This significant difference suggests that the tissues of hypospadias patients themselves, particularly the prepuce commonly used as graft material, may have intrinsic defects.

The choice of graft material further supports this concern. Systematic reviews indicate that buccal mucosa grafts may offer better long-term outcomes compared to preputial skin grafts [6]. Furthermore, a comparative study of Bracka’s two-stage surgery for proximal hypospadias found a lower complication rate with buccal mucosa than with preputial skin (20% vs. 31%), and more satisfactory cosmetic results [7]. These findings collectively suggest that the dysplastic preputial tissue of hypospadias patients is a potential key factor affecting postoperative healing.

Research on the abnormal characteristics of the hypospadic prepuce is increasing. Studies have shown a significant reduction in preputial microvessel density in hypospadias patients, which correlates negatively with severity [8]. Reduced expression of epidermal growth factor receptor (EGFR) in both the inner and outer preputial skin further supports impaired healing potential [9]. While omics technologies have begun to explore its molecular basis, existing studies are limited: gene microarray analysis identified 24 upregulated genes [10]; proteomic studies suggest mild hypospadias is related to mitochondrial energy generation and apoptosis, whereas severe types are associated with complement activation and coagulation cascades [11,12]. These clues all imply that the hypospadic prepuce exists in a unique molecular and cellular microenvironment that is unfavorable for normal repair.

However, most existing research remains at the level of preliminary observation and correlation analysis, failing to integrate these fragmented findings into a coherent pathophysiological process that systematically elucidates the specific mechanisms impairing tissue healing capacity. In particular, the key histological structural changes ultimately caused by these molecular alterations, which directly impact surgical outcomes, remain unclear.

Therefore, this study aims to bridge this knowledge gap. We hypothesize that a series of molecular abnormalities in the hypospadic prepuce collectively drive a specific pathological change, which forms the structural basis for its reduced healing capacity. To test this hypothesis, we employed integrated transcriptomic sequencing and histological analysis to comprehensively map the differences in gene expression profiles between hypospadic and normal prepuce, reveal the core biological processes regulated by these differentially expressed genes (DEGs) through bioinformatics analysis, and ultimately identify corresponding substantial changes at the histological level, thereby providing new and solid evidence for understanding the root causes of high postoperative complication rates in hypospadias.

## 2. Materials and Methods

### 2.1. Patient Enrollment

This study was approved by the Ethics Committee of Shanghai Children’s Hospital (No. 2022R103-F01). All guardians of the children included in this study were fully informed and provided written informed consent. Thirty-one primary hypospadias cases observed in our hospital from December 2023 to August 2025 were included. Based on the position of the urethra after penile straightening, patients were divided into distal hypospadias (coronal groove and subglanular, n = 11), middle hypospadias (penile shaft, n = 9), and proximal hypospadias (scrotum, and perineum, n = 11), aged from 9 months to 79 months (mean age: 34.55 months). Additionally, 21 age-matched children undergoing circumcision for phimosis served as controls, aged from 5 months to 72 months (mean age: 38.52 months). All children in the experimental group had no history of previous hypospadias repair surgery or penile trauma, and no history of topical hormone use. Control samples were obtained from phimosis children without balanoposthitis or preputial trauma history, and who had not received any treatment for local penile lesions.

### 2.2. Sample Collection

Control group: inner preputial tissue from the dorsal 12 o’clock and ventral 6 o’clock positions. Experimental group: inner preputial tissue from the dorsal 12 o’clock position and the ventral prepuce surrounding the urethral meatus. A portion of the surgically removed preputial specimens was immediately frozen in liquid nitrogen, and another portion was fixed in 4% PFA.

### 2.3. Histological Staining

Tissue samples were stored in 4% PFA solution. After embedding in paraffin, these tissues were serially sectioned (5–7 μm). Sections were subjected to H&E staining, Masson’s trichrome staining, Picrosirius red staining, and immunohistochemical analysis to assess Collagen I (Col I), Collagen III (Col III), and CD31.

### 2.4. Transcriptomic Sequencing and Analysis

Total RNA was extracted from cells using TRIzol reagent, and RNA concentration was determined via NanoDrop2000 (Thermo Scientific, Waltham, MA, USA), and the RIN of RNA was determined by an Agilent Bioanalyzer 4150 system (Agilent Technologies, Santa Clara, CA, USA). Only qualified samples will be used for library construction. Fragmentation and purification were then performed using Oligo(dT) beads, followed by synthesis of first-strand and second-strand cDNA. The synthesized double stranded cDNA fragments were then adapter-ligated for preparation of the paired-end library. Adaptor-ligated cDNA were used for PCR amplification. PCR products were purified (AMPure XP system) and library quality was assessed on an Agilent Bioanalyzer 4150 system. Finally, the library preparations were sequenced on an Illumina Novaseq 6000 (or MGISEQ-T7) (Illumina, San Diego, CA, USA) and 150 bp paired-end reads were generated. The data generated from Illumina (or BGI) platform were used for bioinformatics analysis. Gene quantitative expression analysis was achieved by aligning the raw data with the reference sequence using HISAT2 software (version 2.2.2) (http://daehwankimlab.github.io/hisat2/, accessed on 11 November 2025). Differential expression analysis between the two groups was performed using the R package (version 4.5.2) DESeq2, with the significance threshold set at an adjusted *p*-value < 0.05 and |Log2 Fold Change (FC)| ≥ 1.5. Functional annotation of DEGs for Gene Ontology (GO) terms and enrichment analysis of Kyoto Encyclopedia of Genes and Genomes (KEGG) pathways were performed using the clusterProfiler package (version 4.10.0), with a *p*-value < 0.05 set as the significance threshold.

### 2.5. Statistical Analysis

Histological data analysis: ImageJ software (version 1.51) was used for image analysis to calculate the collagen fiber area percentage and vessel density. SPSS 23.0 (SPSS Inc.) was used for statistical analysis. Data are expressed as mean ± standard deviation. Data were tested for normal distribution and homogeneity of variance. If appropriate, unpaired Student’s *t*-test or ANOVA was used. Otherwise, the rank-sum test was used. Fisher’s exact test was used to compare the constituent ratios between the two groups. *p* < 0.05 was considered statistically significant.

## 3. Results

### 3.1. Clinical Characteristics

In total, 21 control and 31 primary hypospadias cases were included. The age and urethral orifice locations in all children with hypospadias and the controls are shown in Table 1.

### 3.2. Gene Expression Profiles

**Figure 1:**
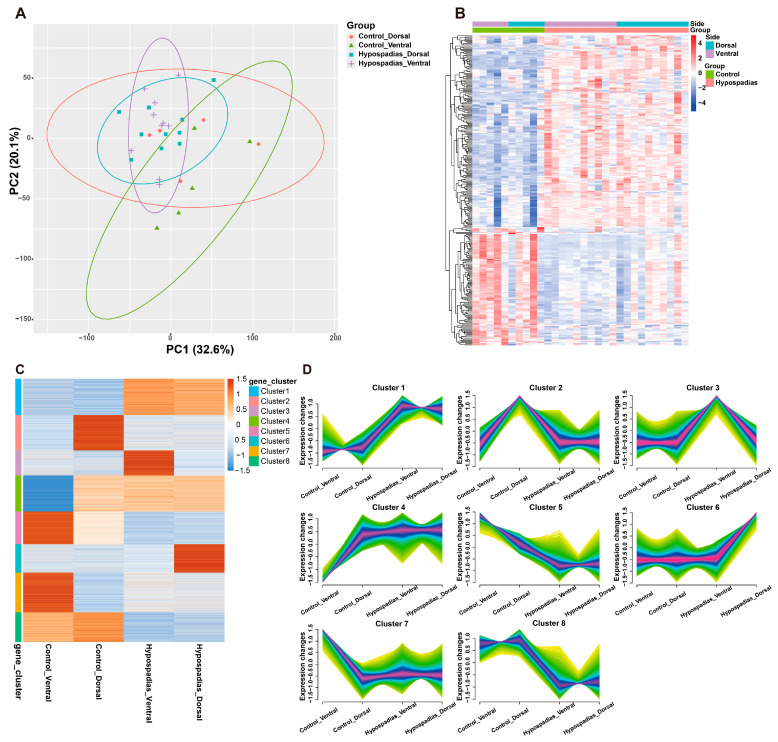
Gene expression in dorsal and ventral inner prepuces between the hypospadias and control group. (**A**) Principal Component Analysis (PCA) plot. (**B**) Heatmap of genes expressed in the different groups. (**C**,**D**) Clustering analysis of the transcriptomic data divided genes into 8 clusters based on expression similarity.

Principal Component Analysis (PCA) plot displayed the overall transcriptomic differences and variances between the hypospadias and control groups, as well as between dorsal and ventral samples. This plot clearly shows that inter-group differences exceed intra-group variations (Figure 1A). Dorsal and ventral inner prepuce samples from hypospadias patients and controls were subjected to transcriptomic sequencing (Figure 1B). Based on expression similarity, clustering analysis of the transcriptomic data grouped the genes into eight distinct clusters (Cluster 1–8) (Figure 1C). Genes in Cluster 1 and Cluster 8 exhibited consistently increased or decreased expression, respectively, in both the dorsal and ventral inner prepuce of hypospadias patients. In contrast, Cluster 3 and Cluster 6 contained genes specifically upregulated in the ventral or dorsal inner prepuce of hypospadias patients, respectively (Figure 1D).

### 3.3. DEGs Between Hypospadias Children and Controls

**Figure 2:**
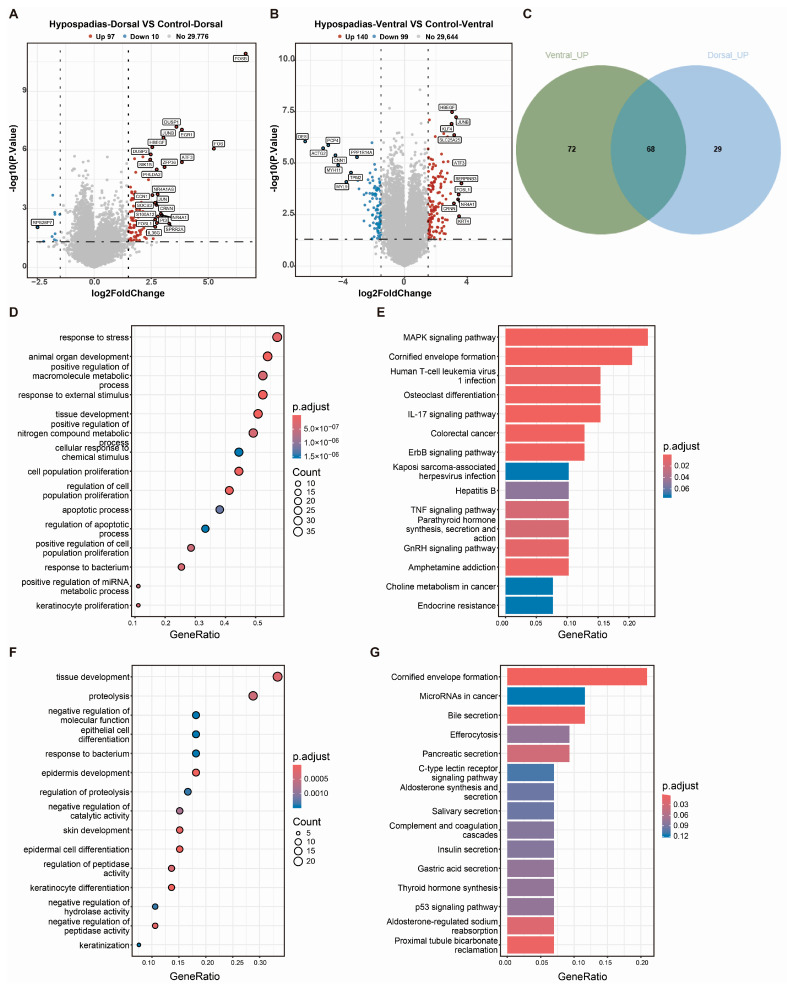
DEGs in the hypospadias group. (**A**) Volcano plots of differentially expressed genes in the dorsal inner prepuce between the hypospadias and control groups. (**B**) Volcano plots of differentially expressed genes in the ventral prepuce between the hypospadias and control groups. (**C**) Venn diagram showing upregulated genes in the ventral and dorsal inner prepuces. (**D**,**E**) GO enrichment and KEGG analysis of Genes co-upregulated in both ventral and dorsal inner prepuces. (**F**,**G**) GO enrichment and KEGG analysis of Genes upregulated in ventral prepuce only.

Comparing gene expression in the dorsal inner prepuce between the hypospadias and control groups (∣log_2_FC∣ ≥ 1.5, *p* < 0.05) identified 107 differentially expressed genes (DEGs), comprising 97 upregulated and 10 downregulated genes in the hypospadias group (Figure 2A). Analysis of the ventral prepuce revealed 140 upregulated and 99 downregulated DEGs in the hypospadias group (Figure 2B). Among these, 68 upregulated DEGs were common to both ventral and dorsal regions of the hypospadias prepuce (Figure 2C). These were primarily enriched in the MAPK signaling pathway, cornified envelope formation, HTLV-I infection, IL-17 signaling pathway, and TNF signaling pathway (Figure 2D,E). Additionally, 72 DEGs were exclusively upregulated in the ventral hypospadias prepuce, with significant enrichment in pathways such as cornified envelope formation, microRNAs in cancer, bile secretion, efferocytosis, C-type lectin receptor signaling pathway, and complement and coagulation cascades (Figure 2C,F,G).

### 3.4. Fibrosis-Related DEGs in Hypospadias

**Figure 3:**
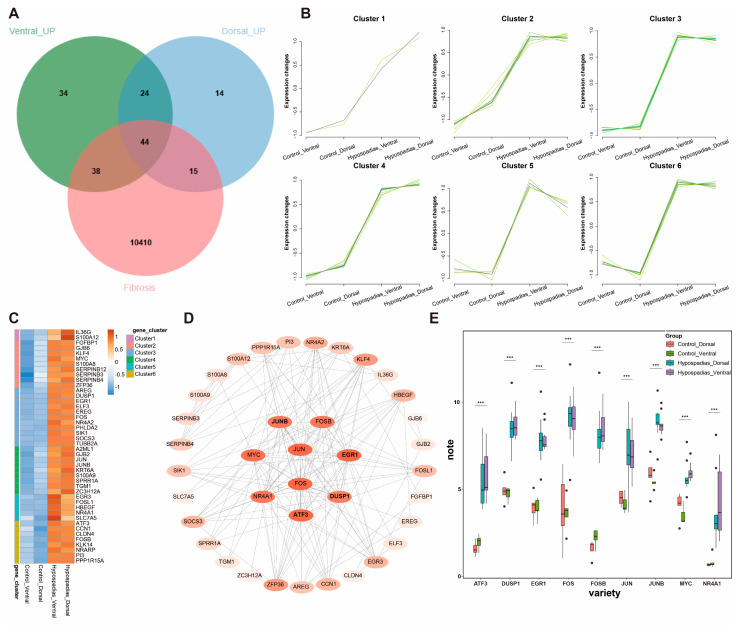
Fibrosis-related DEGs in hypospadias. (**A**) Intersection of fibrosis-related genes and DEGs from the ventral and dorsal prepuces in hypospadias. (**B**) Clustering analysis of the 44 fibrosis-related DEGs in hypospadias (Cluster 1–6). (**C**) Heatmap of fibrosis-related genes expressed in the different groups. (**D**) Protein–protein interaction (PPI) network. (**E**) The expression levels of most significant fibrosis-related DEGs in hypospadias. *** *p* < 0.001.

We retrieved a total of 10,507 fibrosis-associated genes from the GeneCards database. Intersection of the dorsal and ventral DEGs with this fibrosis-related gene set identified 44 fibrosis-associated DEGs in hypospadias (Figure 3A–C). Cluster analysis categorized these genes into six distinct clusters (Clusters 1–6), each representing a group of genes with shared expression patterns (Figure 3B). Protein–protein interaction (PPI) network analysis revealed that the most prominent fibrosis-related DEGs included FOS, JUN, FOSB, JUNB, ATF3, EGR1, MYC, NR4A1, and DUSP1 (Figure 3D). Expression levels of these nine genes were subsequently compared across groups (Figure 3E).

### 3.5. Histological Validation of Preputial Fibrosis

**Figure 4:**
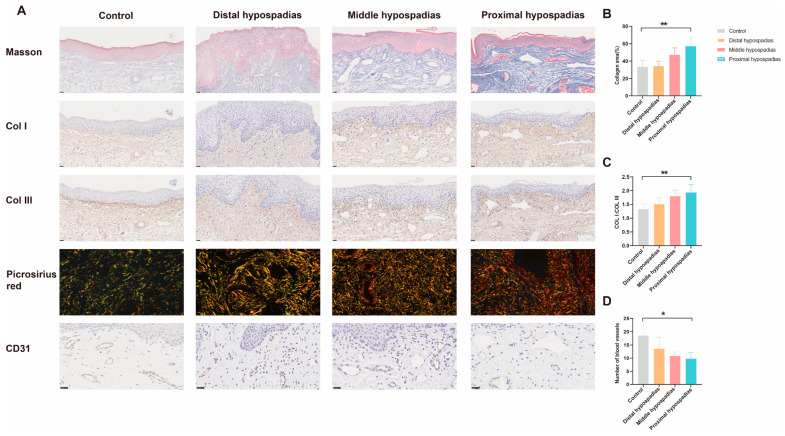
Histological staining of the dorsal inner prepuces in different hypospadias subtypes. (**A**) Masson’s staining, immunohistochemical staining images of Col I, Col III and CD31, as well as picrosirius red polarization image. Magnification: Masson 20×, Col I 20×, Col III 20×, Picrosirius red 40×, CD31 40×. (**B**) Analysis of collagen area% from Masson’s staining. (**C**) The collagen I/III ratio according to picrosirius red polarization image. (**D**) Microvessel density in inner prepuces. Data are expressed as the mean ± standard deviation (SD). * *p* < 0.05, ** *p* < 0.01.

**Figure 5:**
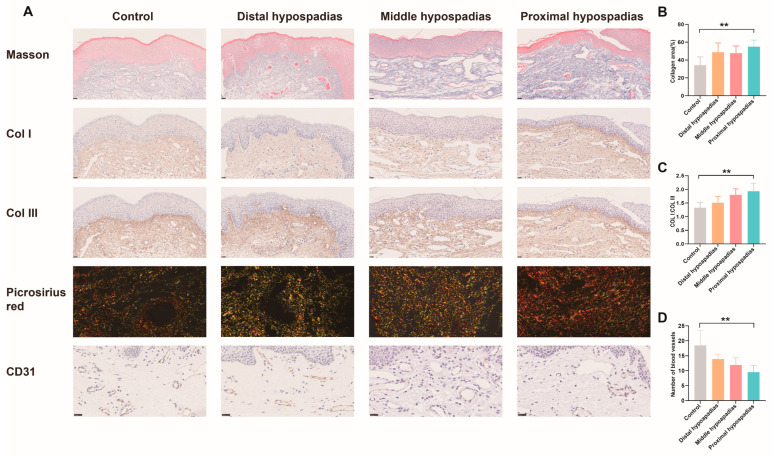
Histological staining of the ventral prepuces in different hypospadias subtypes. (**A**) Masson’s staining, immunohistochemical staining images of Col I, Col III and CD31, as well as picrosirius red polarization image. Magnification: Masson 20×, Col I 20×, Col III 20×, Picrosirius red 40×, CD31 40×. (**B**) Analysis of collagen area according to Masson’s staining. (**C**) The collagen I/III ratio according to picrosirius red polarization image. (**D**) Microvessel density in inner prepuces. Data are expressed as the mean ± standard deviation (SD). ** *p* < 0.01.

Masson’s trichrome staining revealed increased collagen fiber content in the dermis of the dorsal inner prepuce of hypospadias patients compared with controls (Figure 4A). Picrosirius red staining further demonstrated a marked increase in Type I collagen fibers correlating with hypospadias severity (Figure 4A,C). Consistently, immunohistochemical analysis confirmed significantly elevated Type I collagen expression in the inner prepuce of hypospadias patients relative to controls (Figure 4A). CD31 immunohistochemistry indicated reduced microvessel density in the hypospadias group, with a progressive decrease observed as disease severity increased (Figure 4A,D). Analogous findings were obtained from the ventral prepuces, showing consistent trends with the dorsal side (Figure 5).

## 4. Discussion

Through integrated transcriptomic and histological analysis, this study demonstrates that the inner prepuce in hypospadias children exhibits a significant fibrotic phenotype coupled with impaired angiogenesis. Furthermore, we preliminarily delineate the potential molecular regulatory network underlying these pathological features. The main findings include: First, a large number of differentially expressed genes exist in the inner preputial tissue of hypospadias children, 44 of which are closely related to fibrosis, with core genes including FOS, JUN, FOSB, JUNB, ATF3, EGR1, MYC, NR4A1, and DUSP1, most of which are associated with pro-fibrotic and inflammatory pathways such as MAPK, IL-17, and TNF; Second, histological validation confirmed significantly increased collagen deposition, an increased Type I/III collagen ratio, and significantly reduced microvessel density in the hypospadic prepuce, changes which correlated positively with the severity of hypospadias.

Our study is the first to systematically compare tissue differences between hypospadias and normal inner prepuce at the transcriptome level and identified fibrosis as one of the most significant abnormal phenotypes in the prepuce of hypospadias. Previous studies have mostly focused on the urethral plate and surrounding tissues. Da Silva et al. found increased collagen fibers and decreased elastic fibers in the urethral plate of hypospadias children, suggesting the presence of a fibrotic phenotype [13]. Yutaro Hayashi et al. further reported widespread distribution of Type I collagen and absence of Type III collagen in the tissue underlying the urethral plate, potentially affecting penile curvature [14]. Bao xingqi et al. also found histomorphological abnormalities in the corpus spongiosum, correlating with severity [15]. Recently, Zhu et al. reported high expression of HNF-1α in the prepuce of hypospadias promoting fibrosis [16], which aligns well with the upregulation of pro-fibrotic genes found in our study, further supporting the important role of preputial fibrosis in the pathology of hypospadias.

From a mechanistic perspective, the DEGs were significantly enriched in pathways such as MAPK, IL-17, C-type lectin receptor signaling, and complement and coagulation cascades. These pathways intertwine to collectively constitute a pro-fibrotic microenvironment. Notably, genes like FOS, JUN, and FOSB discovered in this study belong to the AP-1 transcription factor complex, which plays a key role in cellular stress and inflammatory responses [17]. Han Xiang et al. found increased c-fos expression in both DEHP-induced hypospadias rat models and human preputial tissue, more pronounced in severe cases [18]. Karabulut et al., via microarray analysis, also found 24 genes upregulated in the prepuce of hypospadias, including DUSP1, EGR1, FOS, JUN [10], consistent with our results. These genes have been reported in fibrotic processes in other organs like skin, lung, and liver, suggesting they may similarly participate in the fibrosis of hypospadic prepuce, thereby affecting postoperative wound healing.

The significantly reduced microvessel density observed in the prepuce of hypospadias further substantiates previous findings [8]. This phenomenon might be related to reduced local androgen receptor expression [19] and downregulated EGFR expression [9], collectively leading to impaired tissue healing potential.

The findings of this study have important clinical implications. First, they provide a direct explanation for the high complication rate after proximal hypospadias repair—the graft material itself is fibrotic and has deficient blood supply. Second, they support prioritizing buccal mucosa or other tissues with richer blood supply and lower fibrotic tendency as graft materials in severe cases, consistent with conclusions from several clinical studies [6,7]. In the future, interventions targeting the abnormal pathways (e.g., anti-fibrotic or pro-angiogenic therapies) may become new strategies for improving surgical outcomes.

The current research endeavors still present certain limitations. The sample size was limited, potentially affecting the statistical power of subgroup analyses. Furthermore, the study primarily accomplished transcriptomic screening and histological validation; specific molecular mechanisms and causal relationships still require confirmation through functional experiments (e.g., gene knockdown/overexpression).

## 5. Conclusions

In conclusion, this study, from an integrative multi-omics perspective, confirms the presence of a significant fibrotic microenvironment in the prepuce of hypospadias, characterized by collagen metabolism imbalance and microvascular scarcity, co-regulated by multiple signaling pathways. These findings not only deepen the understanding of the pathogenesis of hypospadias but also provide a theoretical basis for improving surgical strategies and outcome assessment.

## Figures and Tables

**Table 1:** Baseline Demographics and Clinical Characteristics.

	No.	Age/m (Range)	Rrethral Orifice
**Control**	21	38.52 (5,72)	Normal
**Hypospadias**	31	34.55 (9,79)	
**Distal**	11	43.81 (13–79)	Coronal groove, subglanular
**Middle**	9	25.78 (12–66)	Penile shaft
**Proximal**	11	27.18 (9–56)	Scrotum, and perineum

## Data Availability

All data generated or analyzed during this study are included in this published article. The datasets used and analyzed during the current study are available from the corresponding author upon request.
